# Supplementary material for: Macrophages reprogramming improves immunotherapy of IL-33 in peritoneal metastasis of gastric cancer
Source: EMBO Mol Med. 2024 Jan 18;16(2):251–66. doi: 10.1038/s44321-023-00012-y (PMC10897402; doi:10.1038/s44321-023-00012-y)
Supplement: Supplementary file 1 — Appendix [file 44321_2023_12_MOESM1_ESM.pdf]

Appendix

Macrophages reprogramming improves immunotherapy of IL-33 in peritoneal metastasis of gastric cancer

Table of contents

Appendix Table S1………………………………………… **2**

Appendix Table S2………………………………………… **4**

Appendix Table S3………………………………………… **6**

Appendix Table S4………………………………………… **8**

Appendix Fig S1………………………………………… **9**

Appendix Fig S2………………………………………… **10**

Appendix Fig S3………………………………………… **11**

Appendix Fig S4………………………………………… **13**

Appendix Fig S5………………………………………… **14**

Appendix Fig S6………………………………………… **15**

Appendix Fig S7………………………………………… **17**

Appendix Fig S8………………………………………… **18**

Appendix Fig S9………………………………………… **19**

Appendix Fig S10………………………………………… **20**

Appendix Fig S11………………………………………… **21**

Appendix Tables

Appendix Table S1. Clinicopathological characteristics of gastric cancer patients with low (H-score ≤ 4) or high (H-score > 4) IL-33 expression.

**Variable**	**Low IL-33 expression** ***n*** **= 133 (78.24%)**	**High IL-33 expression** ***n*** **= 37 (21.76%)**	***P***
**Age（years）**			
<65	62 (73.81)	22 (26.19)	
≥65	71 (82.56)	15 (17.44)	0.167
**Gender**			
Male	101 (79.53)	26 (20.47)	
Female	32 (74.42)	11 (25.58)	0.483
**Lauren classification**			
Intestinal type	40 (85.11)	7 (14.89)	
Diffuse type	47 (83.93)	9 (16.07)	
Mixed	46 (68.66)	21 (31.34)	0.050
**8th T stage**			
T2	7 (70.00)	3 (30.00)	
T3	79 (78.22)	22 (21.78)	
T4	47 (79.66)	12 (20.34)	0.791
**8th N stage**			
N0/1	18 (94.74)	1 (5.26)	
N2	39 (72.22)	15 (27.78)	
N3	76 (78.35)	21 (21.65)	0.123
**8th AJCC stage**			
III	125 (78.13)	35 (21.87)	
IV	8 (80.00)	2 (20.00)	1.000
**Vascular invasion**			
Negative (-)	32 (80.00)	8 (20.00)	

Positive (+)	101 (77.69)	29 (22.31)	0.757
**Neural invasion**			
Negative (-)	8 (72.73)	3 (27.27)	
Positive (+)	125 (78.62)	34 (21.38)	0.936
**E-cadherin**			
Negative (-)	18 (90.00)	2 (10.00)	
Positive (+)	115 (76.67)	35 (23.33)	0.285
**PD-L1**			
CPS<10	87 (76.32)	27 (23.68)	
CPS≥10	46 (82.14)	10 (17.86)	0.387

AJCC, American Joint Committee on Cancer; PD-L1, programmed death protein-legend 1; CPS, combinational positive score.

Appendix Table S2. Commercially available antibodies

Antibody	Manufacturer	Catalogue number
In vivomab anti-mouse CSF1R	BioXCell	BE0213
Anti-IL-33	Abcam	ab207737
Anti-NF-κB p65	Cell Signaling Technology	8242
Anti-Phospho-NF-κB p65	Cell Signaling Technology	3033
Anti-p44/42 MAPK	Cell Signaling Technology	4695
Anti-Phospho-p44/42 MAPK	Cell Signaling Technology	4370
Anti-p38 MAPK	Cell Signaling Technology	8690
Anti-Phospho-p38 MAPK	Cell Signaling Technology	4511
Anti-GATA3	Cell Signaling Technology	5852
Anti-GAPDH	Cell Signaling Technology	5174
Anti-PD-L1	Cell Signaling Technology	13684
Anti-E-cadherin	Cell Signaling Technology	14472S
Goat anti-rabbit HRP antibody	Beyotime	A0208
Anti-mouse CD3	BioLegend	100203
Anti-mouse CD4	BioLegend	100407
Anti-mouse CD8	BioLegend	100711
Anti-human/mouse CD11b	BioLegend	101211

Anti-mouse F4/80	BioLegend	123107
Anti-mouse CD80	BioLegend	104713
Anti-mouse CD86	BioLegend	159203
Anti-mouse CD163	BioLegend	156703
Anti-mouse CD206	BioLegend	141707
Anti-mouse CD11c	BioLegend	117305
Anti-mouse I-A/I-E (MHC-II)	BioLegend	107613
Anti-mouse NK1.1	BioLegend	108707
Anti-mouse iNOS monoclonal antibody (CXNFT)	eBioscience	12-5920-82
Anti-mouse IFN-γ	BioLegend	505809
Anti-human CD80	BioLegend	375404
Anti-human CD163	BioLegend	333606
Anti-GATA3	BioLegend	653812

Appendix Table S3. Commercially available reagents

Reagent	Manufacturer	Catalogue number
TRIzol reagent	Invitrogen	15596026
AmfiRivert cDNA Synthesis Platinum Master Mix	GenDEPOT	R5600-100
Power SYBR Green PCR Master Mix	Applied Biosystem	4367659
Puromycin	Thermo Fisher Scientific	A1113802
Leukocyte Activation Cocktail	BD Bioscience	550583
Fixation/Permeabilization Solution Kit	BD Biosciences	554714
M-CSF	PeproTech	315-02
LPS	Sigma-Aldrich	L2630
Multi-Analyte Flow Assay Kit	BioLegend	740750
JSH-23	MedChemExpress	HY-13982
U0126	MedChemExpress	HY-12031A
SB203580	MedChemExpress	HY-10256
Radio immunoprecipitation	MACKLIN	R874810
BCA protein quantification kit	Vazyme	E112-01
DAPI	Solarbio	C0065
Phalloidin-TRITC	Sigma-Aldrich	P1951

OKT3	eBioscience	14-0037-82
Pan Monocyte Isolation Kit	Miltenyi	130-096-537
CFSE proliferation kit	ThermoFisher Scientific	C34554
Recombinant murine IL-4	PeproTech	214-14
Recombinant murine IL-10	PeproTech	210-10
Recombinant murine IL-33	PeproTech	210-33
Recombinant human IL-2	PeproTech	200-02
Recombinant human IL-4	PeproTech	200-04
Recombinant human IL-13	PeproTech	200-13
Recombinant human IL-15	PeproTech	200-15
Recombinant human IFN-γ	PeproTech	300-02

.

Appendix Table S4. Oligonucleotide primers for qRT-PCR.

Gene name	Forward primer	Reverse primer
GAPDH	GAACGGGAAGCTCACTGG	GCCTGCTTCACCACCTTCT
Il-33	TTATCATAAGGCCAGAGCGG	GAACACAGCAAGCAAAGCCT
Il-6	AGACAGCCACTCACCTCTTCAG	TTCTGCCAGTGCCTCTTTGCTG
Il-1β	CCACAGACCTTCCAGGAGAATG	GTGCAGTTCAGTGATCGTACAGG
Tnf-α	CCTGCTGCACTTTGGAGTGA	TCGAGAAGATGATCTGACTGCC
Tgf-β	TACCTGAACCCGTGTTGCTCTC	GTTGCTGAGGTATCGCCAGGAA
Arg1	ACAGTTTGGCAATTGGAAGCA	CACCCAGATGACTCCAAGATCAG
Cd80	CTCTTGGTGCTGGCTGGTCTTT	GCCAGTAGATGCGAGTTTGTGC
Cd86	CCATCAGCTTGTCTGTTTCATTCC	GCTGTAATCCAAGGAATGTGGTC
Cd163	CCAGAAGGAACTTGTAGCCACAG	CAGGCACCAAGCGTTTTGAGCT

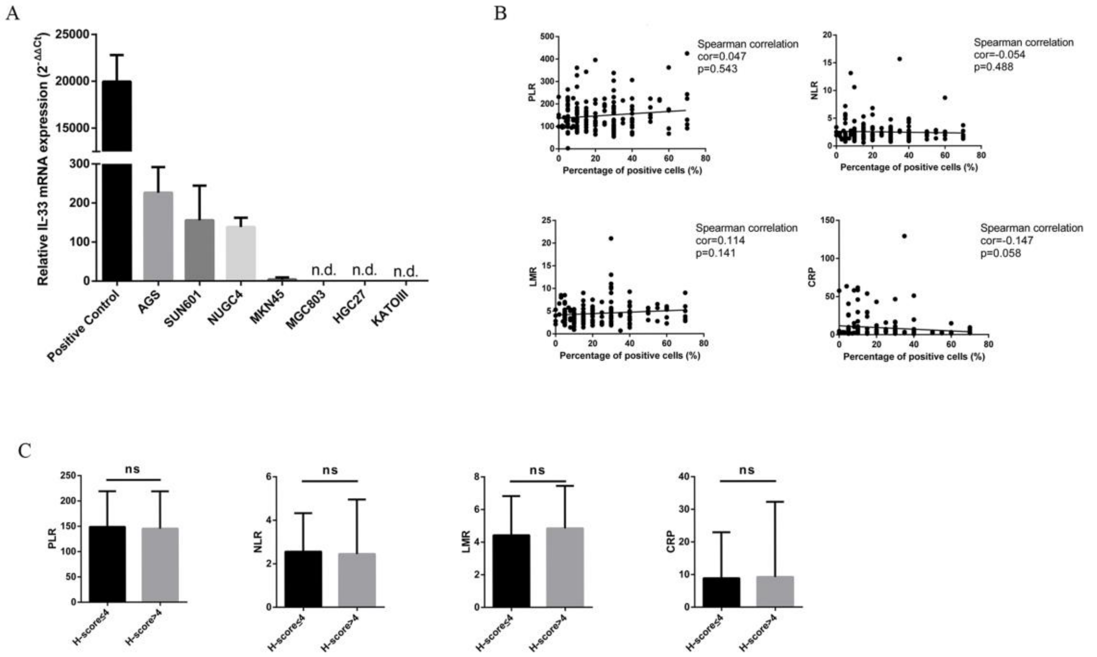

Appendix Fig S1. Supplementary clinical data of IL-33 analysis in GC patients (Related to Fig 1).

(A) Relative *IL*-33 mRNA expression (normal gastric tissues as control) among human GC cell lines were detected by qPCR. Each type of cell line was a representative experiment of at least three independent biological replicates. **(B)** The correlation between blood inflammatory markers (PLR, NLR, LMR and CRP) and the percentage of IL-33 positive cells in tumor tissues of GC patients. **(C)** The correlation between blood inflammatory markers (PLR, NLR, LMR and CRP) and the H-score of IL-33 in tumor tissues of GC patients.

Data information: Data with error bars are shown as mean ± SD. ns, not significant as determined by two-tailed unpaired-sample Student *t* test. n.d., not detected. Also, see Fig 1.

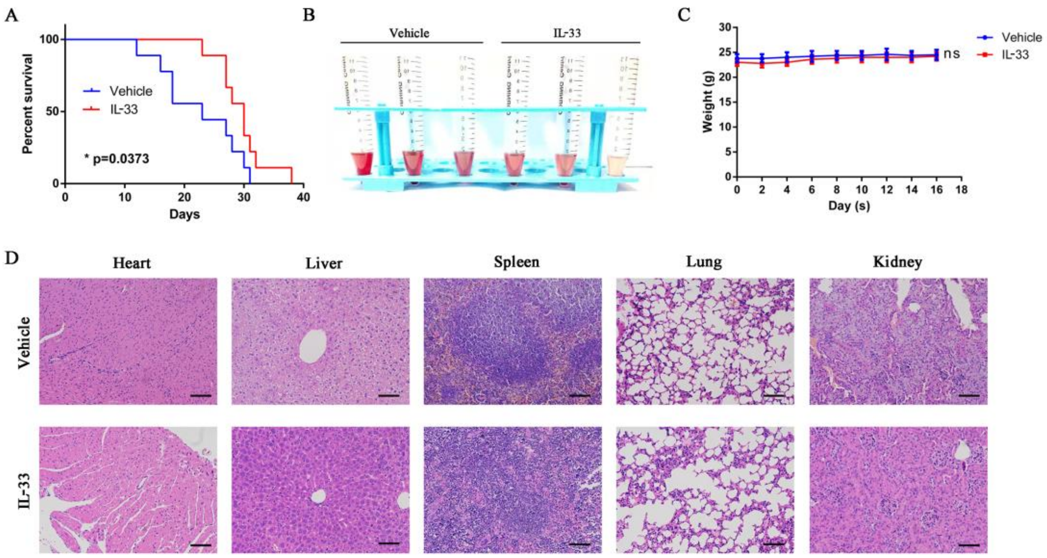

Appendix Fig S2. Survival analysis and safety evaluation of IL-33 intraperitoneal administration in murine GC model (Related to Fig 2).

(A) Survival plot of mice bearing intraperitoneal MFC tumors treated intraperitoneally with PBS or IL-33 every other day after tumor challenge (*n* = 9 per group). **(B)** Ascites of mice bearing intraperitoneal MFC tumors treated intraperitoneally with PBS or IL-33 every other day, harvested at day 16 (*n* = 3 mice per group; 3 independent experiments). **(C)** Body weight of MFC-challenged 615-line mice (*n* = 5 per group) treated with PBS or IL-33 intraperitoneally. **(D)** Safety evaluation of IL-33 in mouse organs of MFC-challenged 615-line mice shown by H&E staining. Scale bar, 100μm.

Data information: Data with error bars are shown as mean ± SD. ns, not significant; $^{*}P < 0.05$ as determined by log-rank test and two-tailed unpaired-sample Student *t* test. Also, see Fig 2.

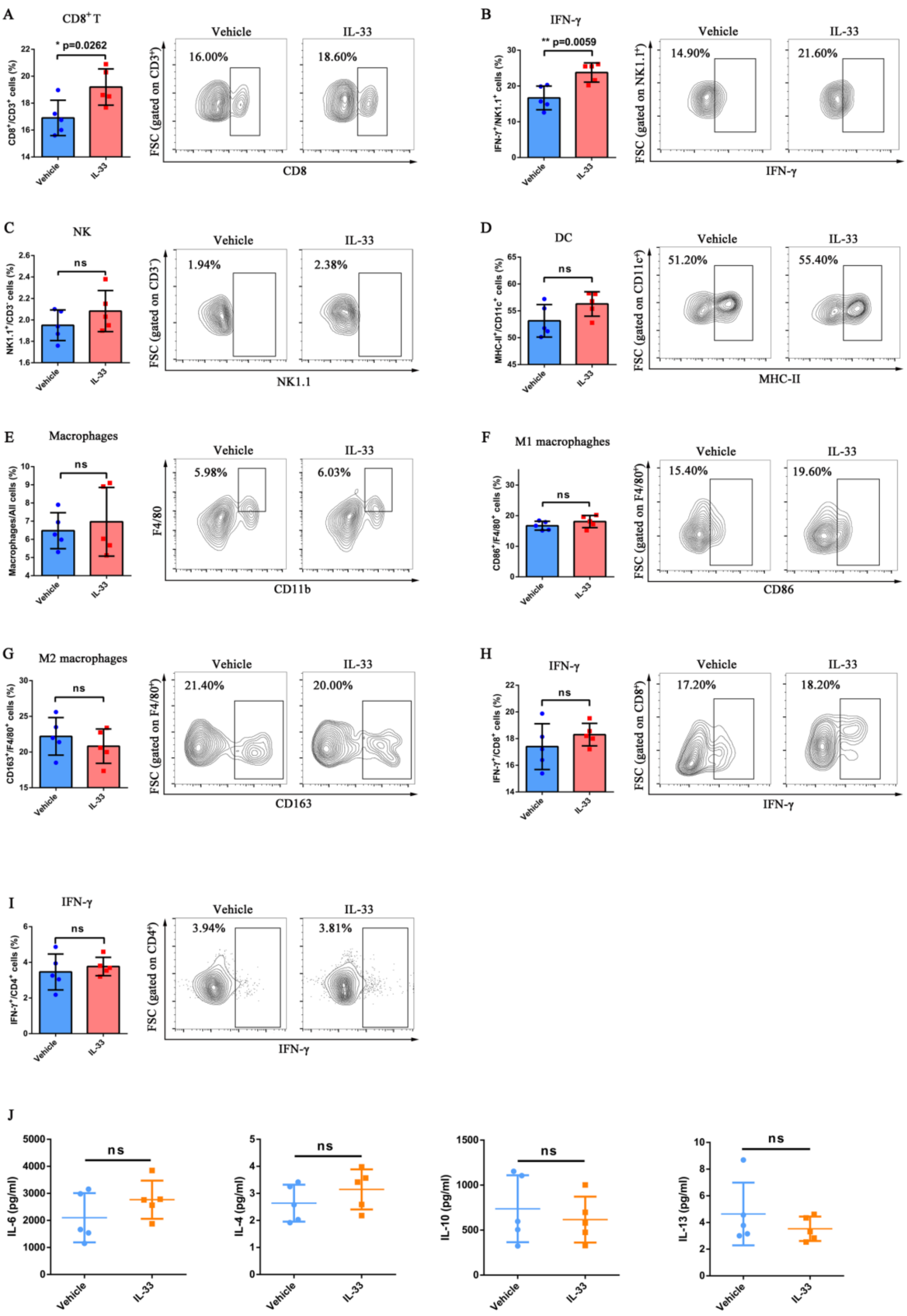

Appendix Fig S3. Intraperitoneal administration of IL-33 changes the immunocytes subgroups in spleen. (Related to Fig 2)

615-line mice (n = 5 per group) developing abdominal dissemination tumors upon intraperitoneal

injection of MFC were treated with PBS or IL-33 intraperitoneally. Mice were sacrificed at treatment endpoint and spleens were removed for analysis. **(A)** Proportions of $CD8^+/CD3^+$ cells in spleen were determined by flow cytometry (*n* = 5 biological replicates). **(B)** Proportions of IFN-γ^+/NK1.1^+ cells in spleen were determined by flow cytometry (*n* = 5 biological replicates). **(C)** Proportions of NK1.1^+/$CD3^-$ cells in spleen were determined by flow cytometry (*n* = 5 biological replicates). **(D)** Proportions of MHC-II^+/$CD11c^+$ cells in spleen were determined by flow cytometry (*n* = 5 biological replicates). **(E)** Proportions of macrophages/all cells in spleen were determined by flow cytometry (*n* = 5 biological replicates). **(F)** Proportions of $CD86^+$/F4/80^+ macrophages in spleen were determined by flow cytometry (*n* = 5 biological replicates). **(G)** Proportions of $CD163^+$/F4/80^+ macrophages in spleen were determined by flow cytometry (*n* = 5 biological replicates). **(H)** Proportions of IFN-γ^+/$CD8^+$ cells in spleen were determined by flow cytometry (*n* = 5 biological replicates). **(I)** Proportions of IFN-γ^+/$CD4^+$ cells in spleen were determined by flow cytometry (*n* = 5 biological replicates). **(J)** Expression of IL-6, IL-4, IL-10 and IL-13 in ascites of abdominal dissemination mouse model was detected using CBA (*n* = 5 biological replicates). Data information: Data with error bars are shown as mean ± SD. ns, not significant; $^*P < 0.05$, $^{**}P < 0.01$ as determined by two-tailed unpaired-sample Student *t* test. Also, see Fig 2.

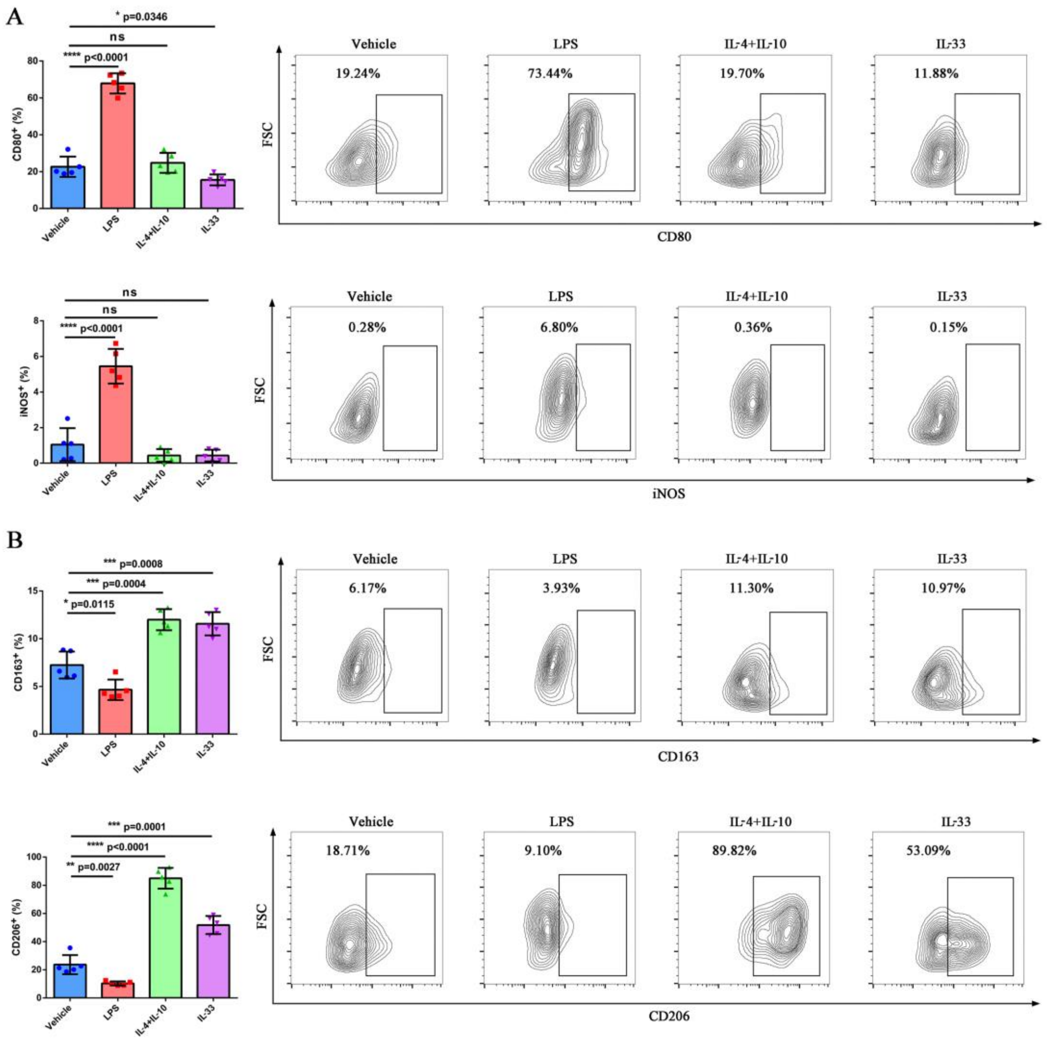

Appendix Fig S4. IL-33 directly triggers the polarization of BMDMs. (Related to Fig 4)

(A) 615-line BMDMs were stimulated with PBS, LPS, IL-4+IL-10 or IL-33 for 48 h, respectively. The proportions of $CD80^+$ and $iNOS^+$ BMDMs were determined by flow cytometry ($n = 5$ biological replicates). **(B)** Proportions of $CD163^+$ and $CD206^+$ BMDMs were determined by flow cytometry ($n = 5$ biological replicates).

Data information: Data with error bars are shown as mean ± SD. ns, not significant; $^{*}P < 0.05$, $^{**}P < 0.01$, $^{***}P < 0.001$, $^{****}P < 0.0001$ by two-tailed unpaired-sample Student t test. Also, see Fig 4.

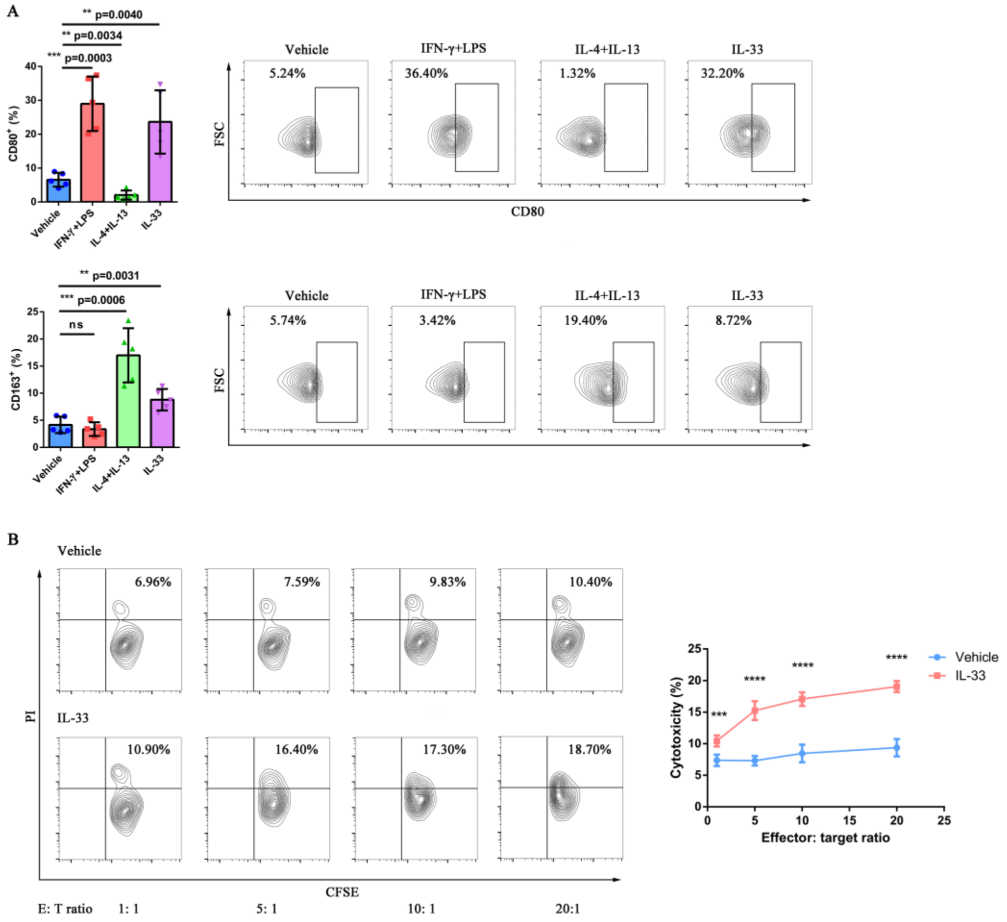

Appendix Fig S5. IL-33 directly triggers the polarization of macrophages from PBMCs and is critical for efficient PBMCs killing. (Related to Fig 4)

(A) Macrophages from PBMCs of GC patients were stimulated by PBS, IFN-γ+LPS, IL-4+IL-13 or IL-33 for 48 h, respectively. Proportions of $CD80^+$ and $CD163^+$ macrophages were determined by flow cytometry (n = 5 biological replicates). **(B)** Apoptotic percentage of SNU601 cells after treatment with vehicle or IL-33 stimulated human PBMCs for 12 h, as determined by flow cytometry (n = 5 biological replicates).

Data information: Data with error bars are shown as mean ± SD. ns, not significant; $^{**}P < 0.01$, $^{***}P < 0.001$, $^{****}P < 0.0001$ by two-tailed unpaired-sample Student t test. Also, see Fig 4.

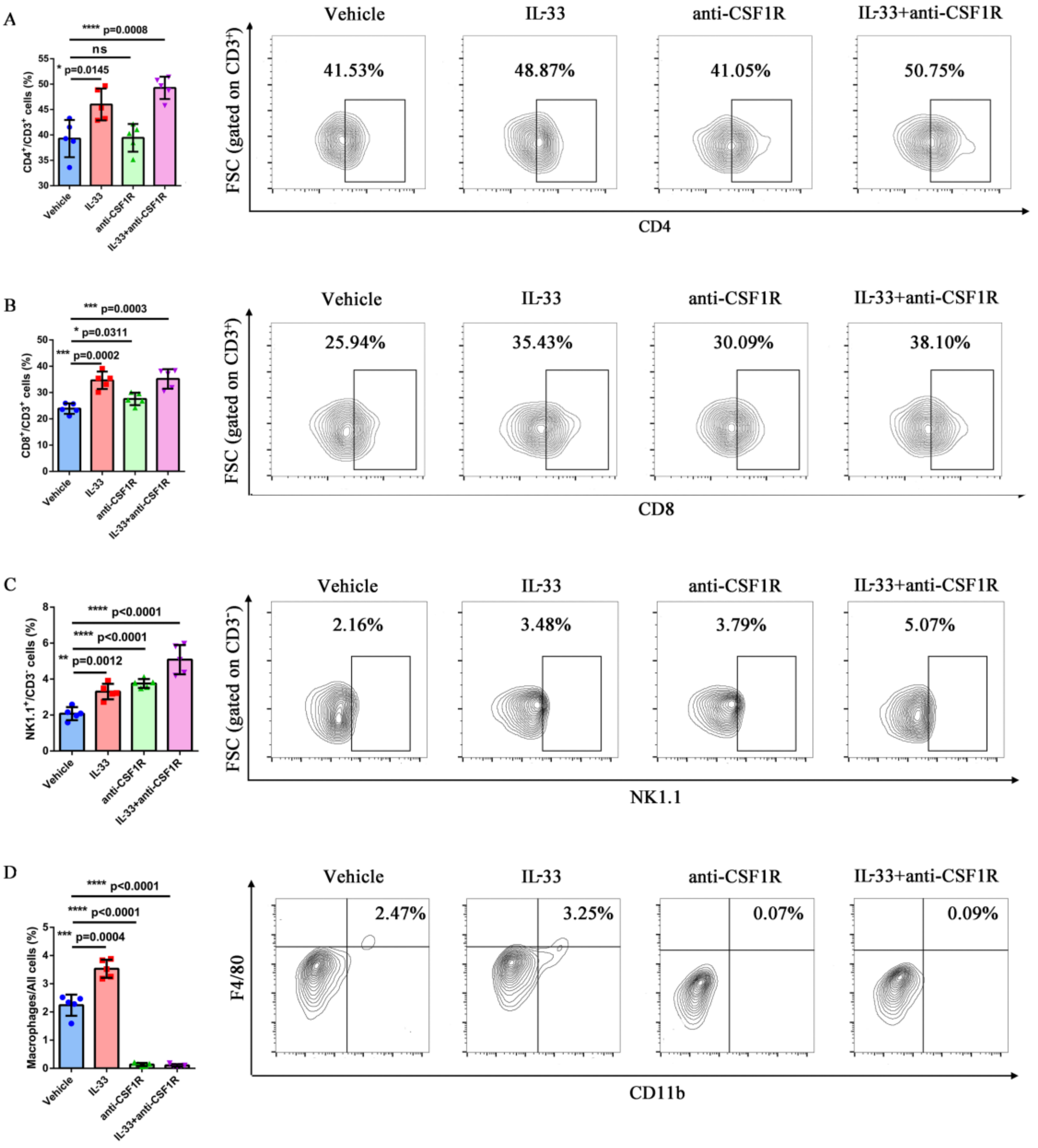

Appendix Fig S6. Combination of IL-33 and anti–CSF1R promotes the infiltration of peritoneal immunocytes in MFC-Luc challenged 615-line mice (Related to Fig 5).

615-line mice (*n* = 5 per group) developing abdominal dissemination tumors upon intraperitoneal injection of MFC-Luc were treated with vehicle, IL-33, anti-CSF1R or IL-33+anti-CSF1R intraperitoneally. Vehicle-treated mice served as controls. **(A)** Proportions of $CD4^+/CD3^+$ cells in abdominal tumors were determined by flow cytometry (*n* = 5 biological replicates). **(B)** Proportions of $CD8^+/CD3^+$ cells in abdominal tumors were determined by flow cytometry (*n* = 5 biological replicates). **(C)** Proportions of $NK1.1^+/CD3^-$ cells in abdominal tumors were determined by flow

cytometry (n = 5 biological replicates). **(D)** Proportions of macrophages/all cells in abdominal tumors were determined by flow cytometry (n = 5 biological replicates).

Data information: Data with error bars are shown as mean ± SD. ns, not significant; $^{*}P < 0.05$, $^{**}P < 0.01$, $^{***}P < 0.001$, $^{****}P < 0.0001$ by two-tailed unpaired-sample Student t test. Also, see Fig 5.

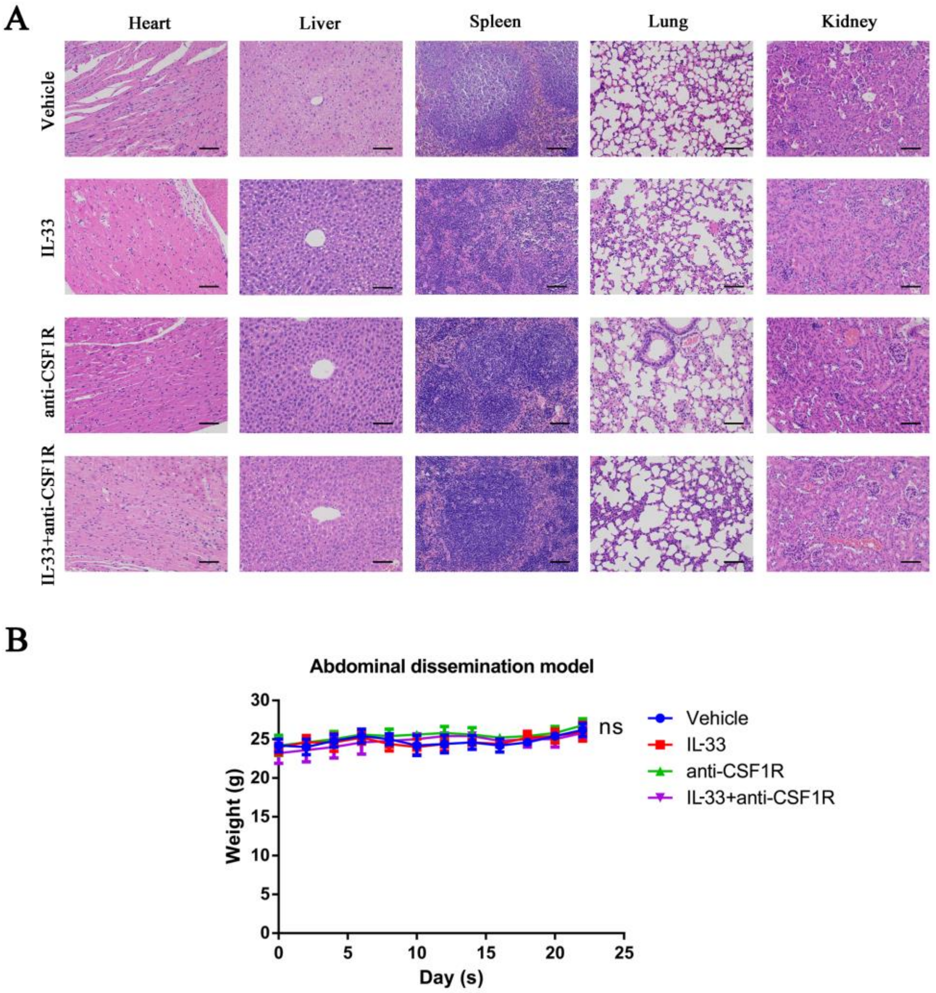

Appendix Fig S7. Safety evaluation of IL-33 combined with anti-CSF1R intraperitoneal administration in murine GC model (Related to Fig 5).

(A) Safety evaluation of IL-33 combined with anti-CSF1R in mouse organs of MFC-Luc-challenged 615-line mice shown by H&E staining. Scale bar, 100μm. **(B)** Body weight of MFC-Luc-challenged 615-line mice (n = 5 biological replicates) treated with vehicle, IL-33, anti-CSF1R or IL-33+anti-CSF1R. Vehicle-treated mice served as controls.

Data information: Data with error bars are shown as mean ± SD. ns, not significant as determined by two-tailed unpaired-sample Student t test. Also, see Fig 5.

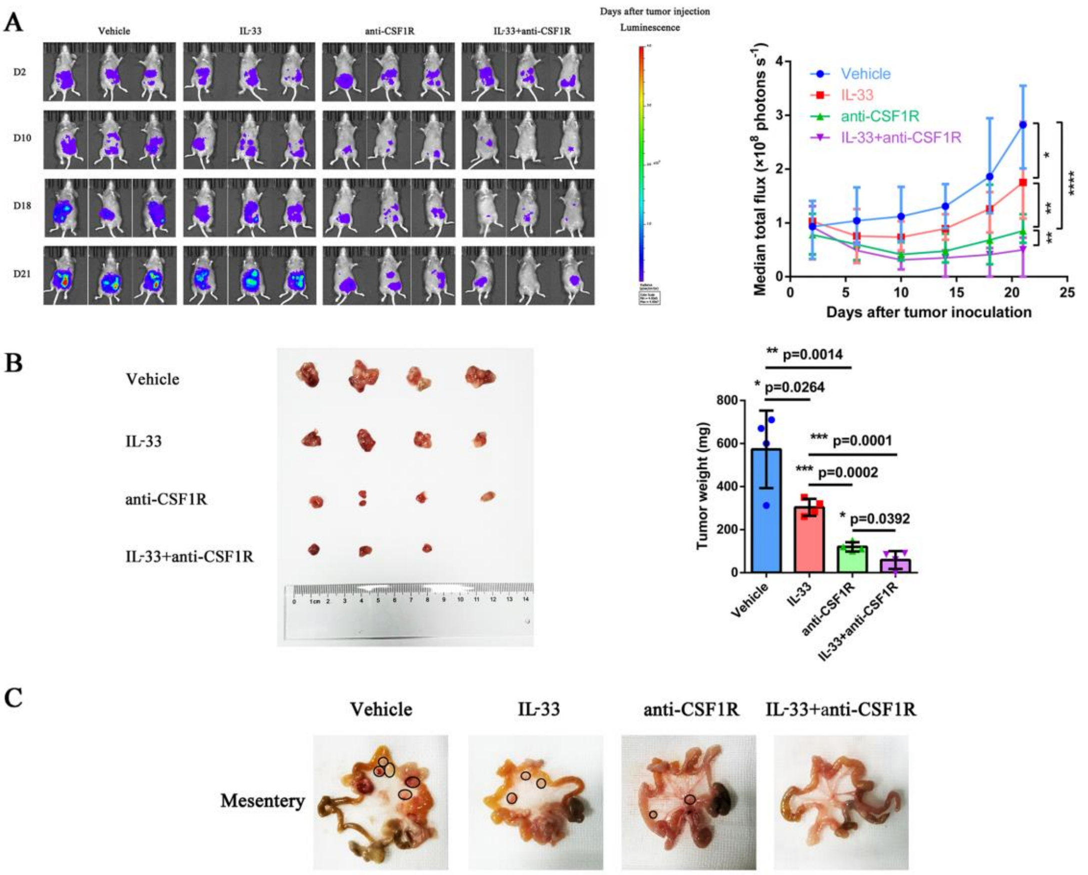

Appendix Fig S8. Pre-administration of anti-CSF1R combined with IL-33 has a robust reduction for abdominal dissemination tumors.

(A) 615-line mice (n = 4 per group) developing abdominal dissemination tumors upon intraperitoneal injection of MFC-Luc were treated with vehicle, IL-33, anti-CSF1R (D-10) or IL-33+anti-CSF1R (D-10). Vehicle-treated mice served as controls. Tumor growth was determined by BLI as photons per second. **(B)** Abdominal dissemination tumors and tumor weight of MFC-Luc-challenged 615 mice treated with vehicle, IL-33, anti-CSF1R (D-10) or IL-33+anti-CSF1R (D-10) (n = 4 biological replicates). **(C)** Mesenteric dissemination tumors of MFC-Luc-challenged 615 mice treated with vehicle, IL-33, anti-CSF1R (D-10) or IL-33+anti-CSF1R (D-10).

Data information: Data with error bars are shown as mean ± SD. $^{*}P < 0.05$, $^{**}P < 0.01$, $^{***}P < 0.001$, $^{****}P < 0.0001$ by two-tailed unpaired-sample Student t test.

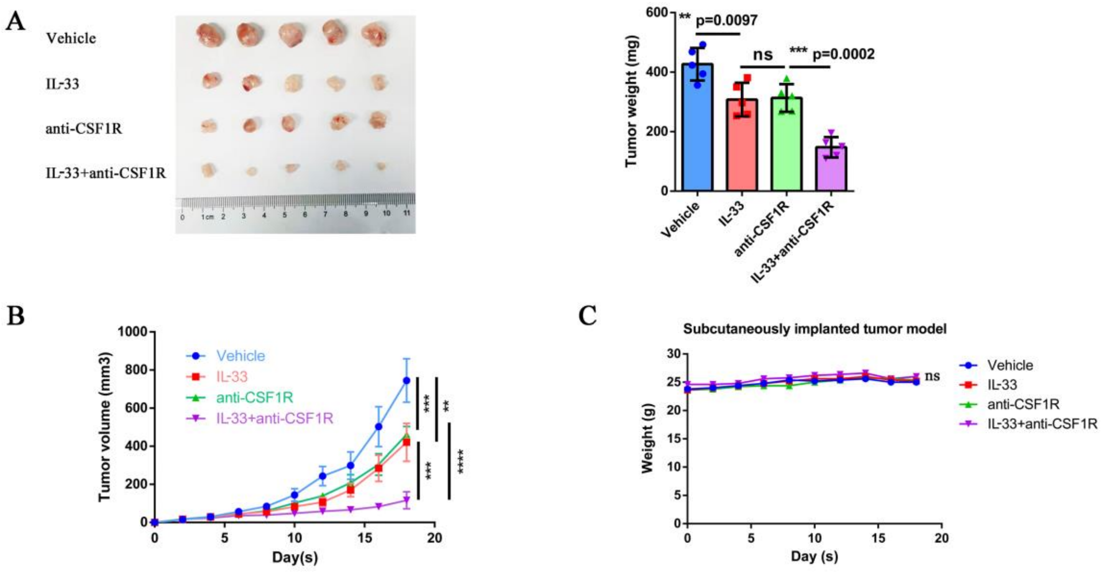

Appendix Fig S9. Combination of IL-33 and anti–CSF1R augments anti-tumor response in the MFC subcutaneous tumor model.

615-line mice (n = 5 per group) developing subcutaneous tumors upon subcutaneous injection of MFC were treated with vehicle, IL-33, anti-CSF1R or IL-33+anti-CSF1R. Vehicle-treated mice served as controls. **(A)** Mice were sacrificed at treatment endpoint and tumors were removed. Tumor weight of MFC-challenged 615 mice treated with vehicle, IL-33, anti-CSF1R or IL-33+anti-CSF1R (n = 5 biological replicates). **(B)** Tumor volumes of MFC-challenged 615 mice treated with vehicle, IL-33, anti-CSF1R or IL-33+anti-CSF1R (n = 5 biological replicates). **(C)** Body weight of MFC-challenged 615-line mice treated with vehicle, IL-33, anti-CSF1R or IL-33+anti-CSF1R (n = 5 biological replicates).

Data information: Data with error bars are shown as mean ± SD. ns, not significant; $^{**}P < 0.01$, $^{***}P < 0.001$, $^{****}P < 0.0001$ by two-tailed unpaired-sample Student t test.

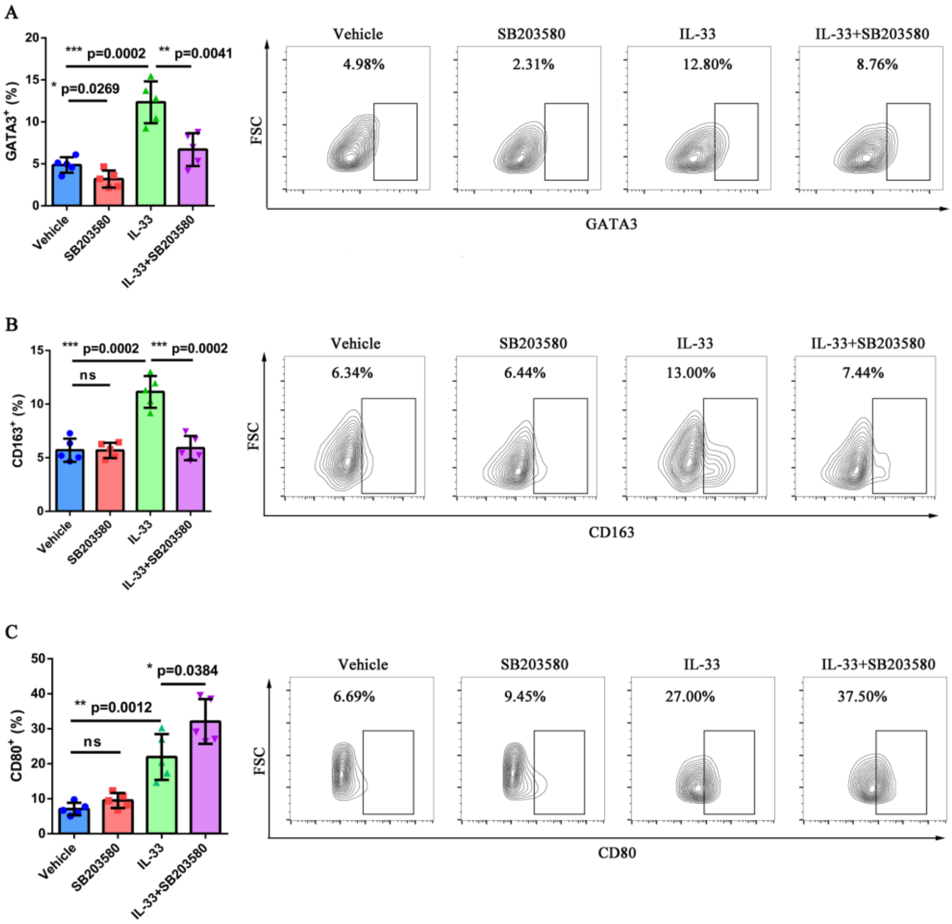

Appendix Fig S10. IL-33 activates the p38-GATA3 signaling pathway to induce M2 polarization of macrophages from GC patients. (Related to Fig 6)

Macrophages from GC patients were pretreated with or without p38 specific inhibitor for 1 h before stimulated with IL-33 or PBS. PBS-treated cells served as controls. **(A)** Expression of GATA3 in IL-33-induced macrophages altered by p38 specific inhibitor (n = 5 biological replicates). **(B)** Expression of CD163 in IL-33-induced macrophages altered by p38 specific inhibitor (n = 5 biological replicates). **(C)** Expression of CD80 in IL-33-induced macrophages altered by p38 specific inhibitor (n = 5 biological replicates).

Data information: Data with error bars are shown as mean ± SD. ns, not significant; $^{*}P < 0.05$, $^{**}P < 0.01$, $^{***}P < 0.001$ by two-tailed unpaired-sample Student t test. Also, see Fig 6.

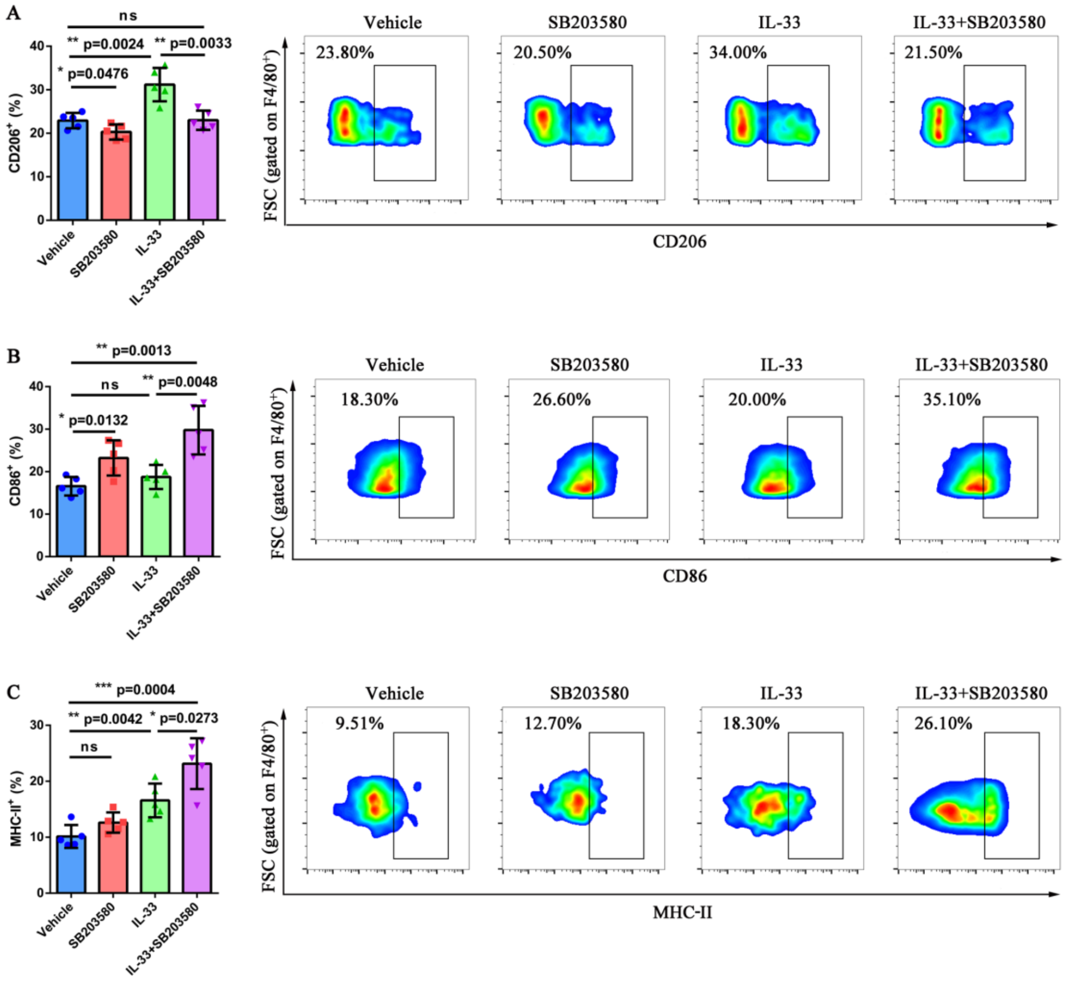

Appendix Fig S11. P38 signaling pathway inhibitor blocks the M2 polarization induced by IL-33. (Related to Fig 7)

615-line mice (n = 5 per group) developing abdominal dissemination upon intraperitoneal injection of MFC were treated with vehicle, SB203580, IL-33 or IL-33+SB203580. Vehicle-treated mice served as controls. **(A)** Proportions of $CD206^+/F4/80^+$ macrophages in abdominal tumors were determined by flow cytometry (n = 5 biological replicates). **(B)** Proportions of $CD86^+/F4/80^+$ macrophages in abdominal tumors were determined by flow cytometry (n = 5 biological replicates). **(C)** Proportions of $MHC\text{-}II^+/F4/80^+$ macrophages in abdominal tumors were determined by flow cytometry (n = 5 biological replicates).

Data information: Data with error bars are shown as mean ± SD. ns, not significant; $^{*}P < 0.05$, $^{**}P < 0.01$, $^{***}P < 0.001$ by two-tailed unpaired-sample Student t test. Also, see Fig 7.
