# Supplementary figures and images for: Macrophages reprogramming improves immunotherapy of IL-33 in peritoneal metastasis of gastric cancer
Source: EMBO Mol Med. 2024 Jan 18;16(2):251–66. doi: 10.1038/s44321-023-00012-y (PMC10897402; doi:10.1038/s44321-023-00012-y)

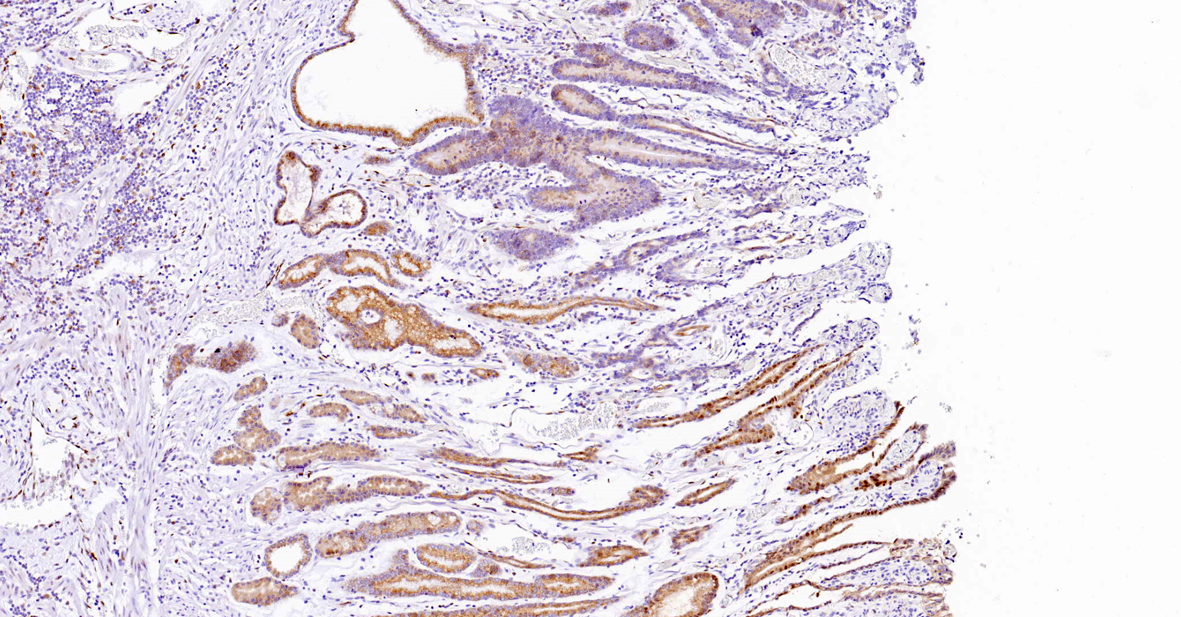

Supplement: Supplementary file 4 — Source Data Fig. 1 [file 44321_2023_12_MOESM4_ESM.zip › Figure 1/1D/1D-IL-33 IHC tumor tissue vs normal tissue.tif]

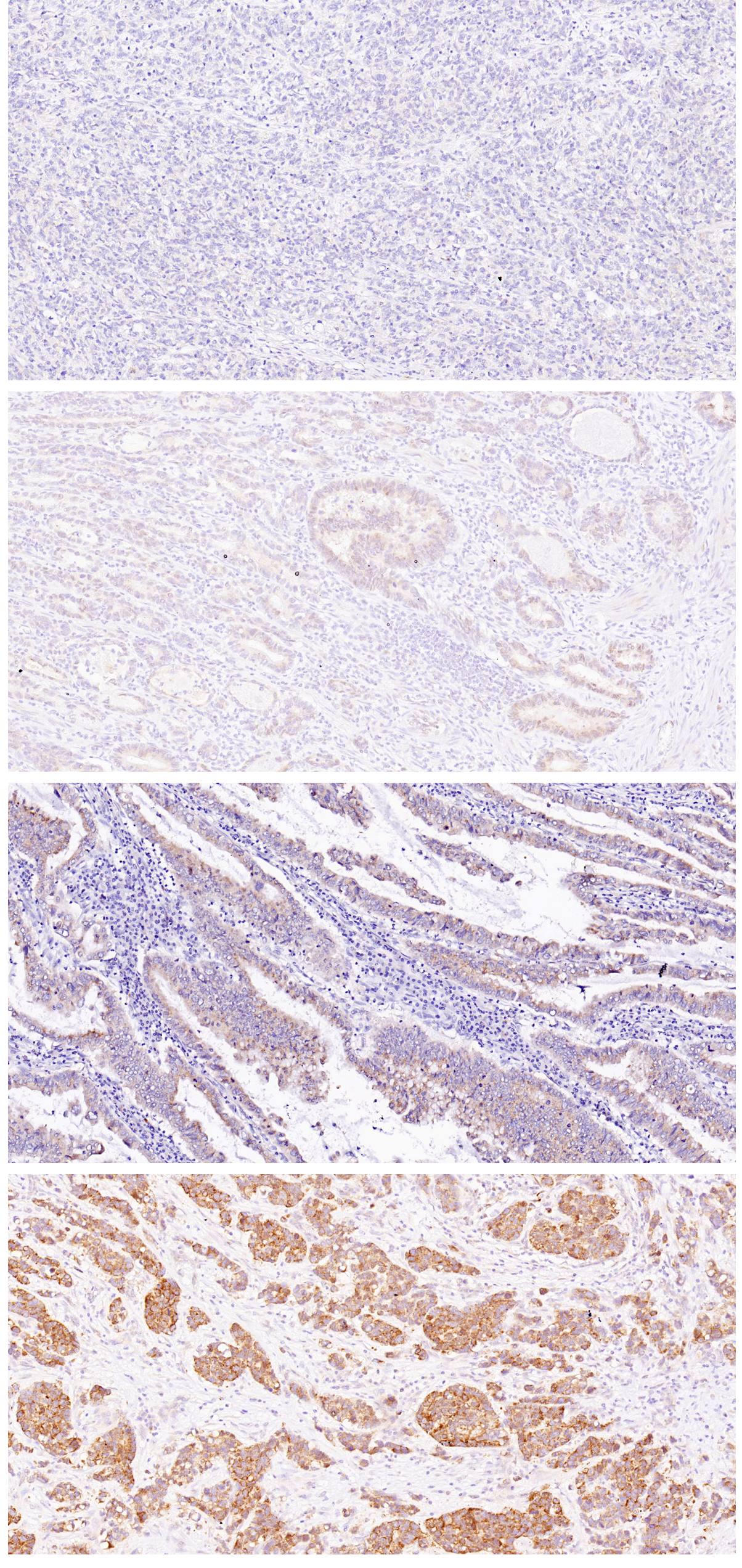

Supplement: Supplementary file 4 — Source Data Fig. 1 [file 44321_2023_12_MOESM4_ESM.zip › Figure 1/1C/1C-IL-33 IHC H-score.tif]

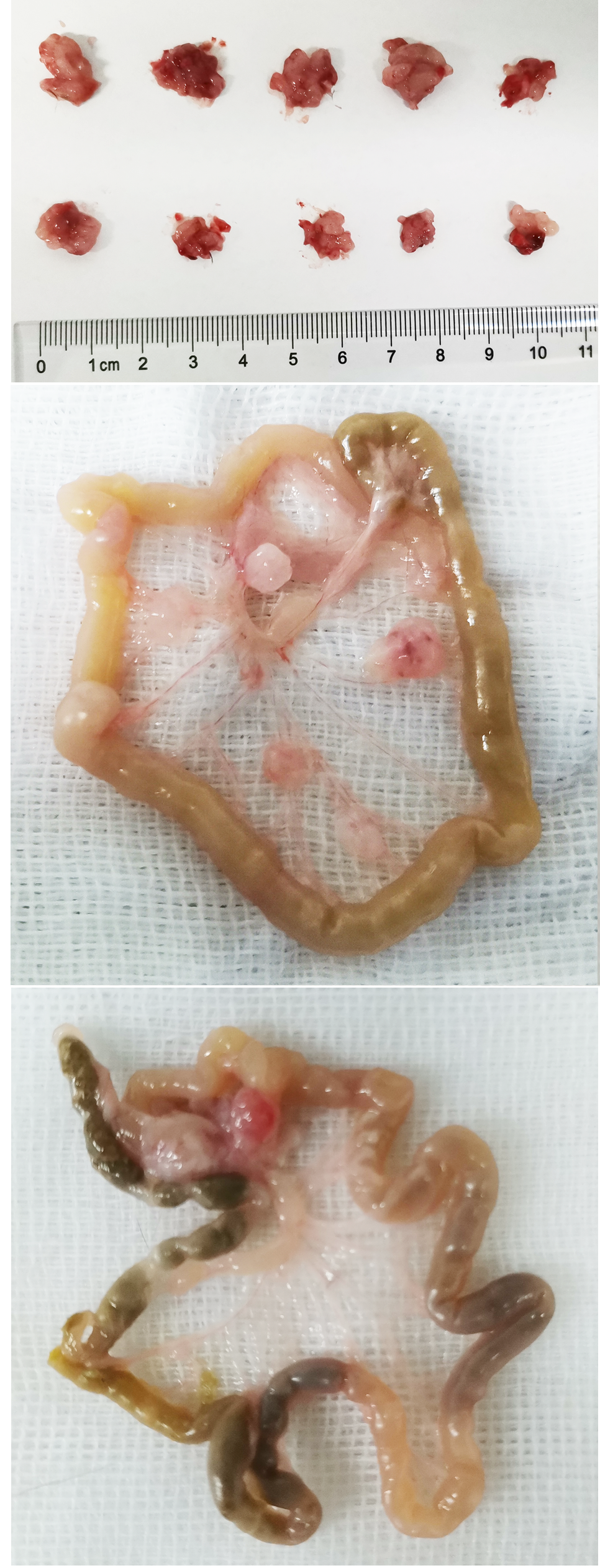

Supplement: Supplementary file 5 — Source Data Fig. 2 [file 44321_2023_12_MOESM5_ESM.zip › Figure 2/2B/2B-Whole mount.tif]

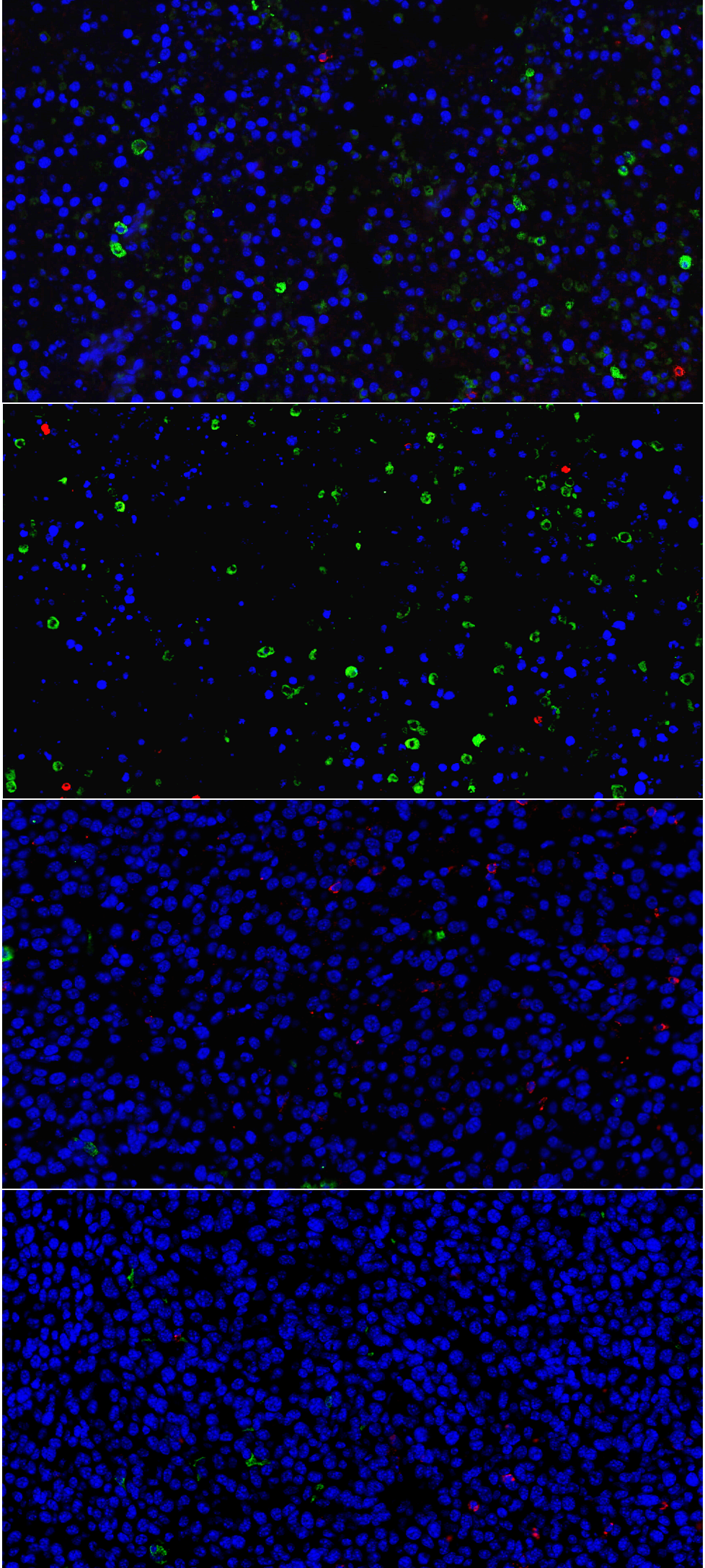

Supplement: Supplementary file 8 — Source Data Fig. 5 [file 44321_2023_12_MOESM8_ESM.zip › Figure 5/5F/5F-Immunofluorescence.tif]

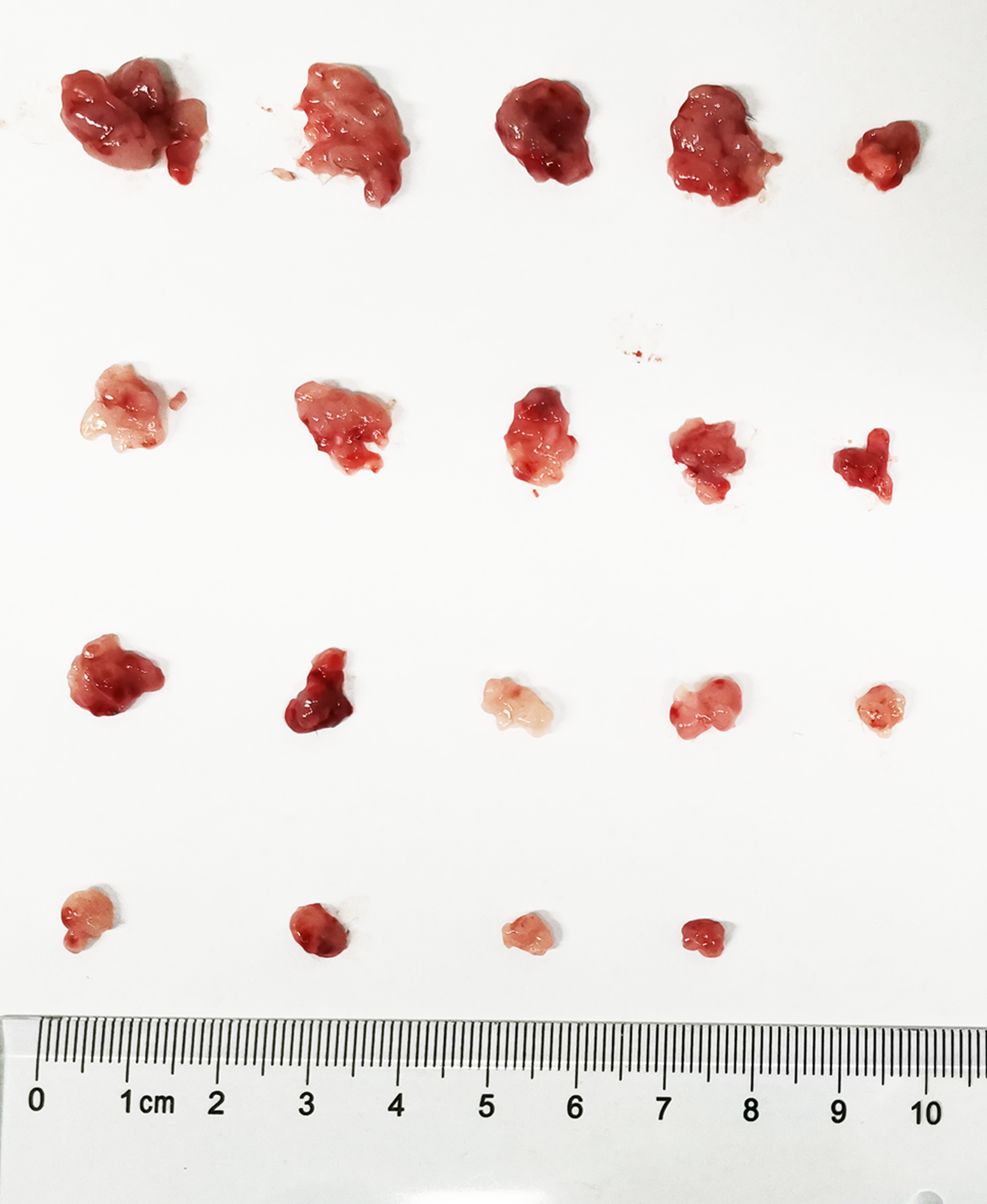

Supplement: Supplementary file 8 — Source Data Fig. 5 [file 44321_2023_12_MOESM8_ESM.zip › Figure 5/5D/5D-Abdominal dissemination tumors.tif]

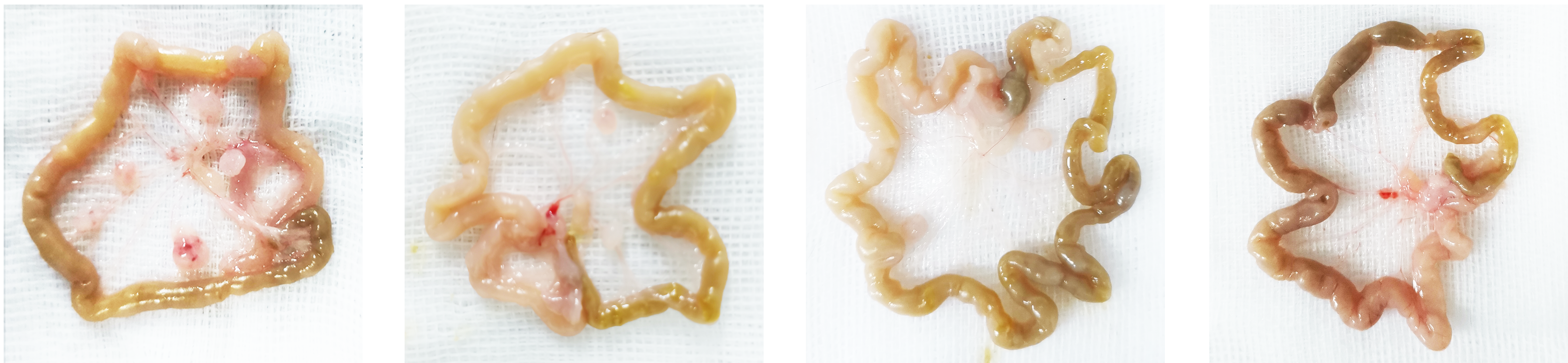

Supplement: Supplementary file 8 — Source Data Fig. 5 [file 44321_2023_12_MOESM8_ESM.zip › Figure 5/5E/5E-Mesenteric dissemination tumors.tif]

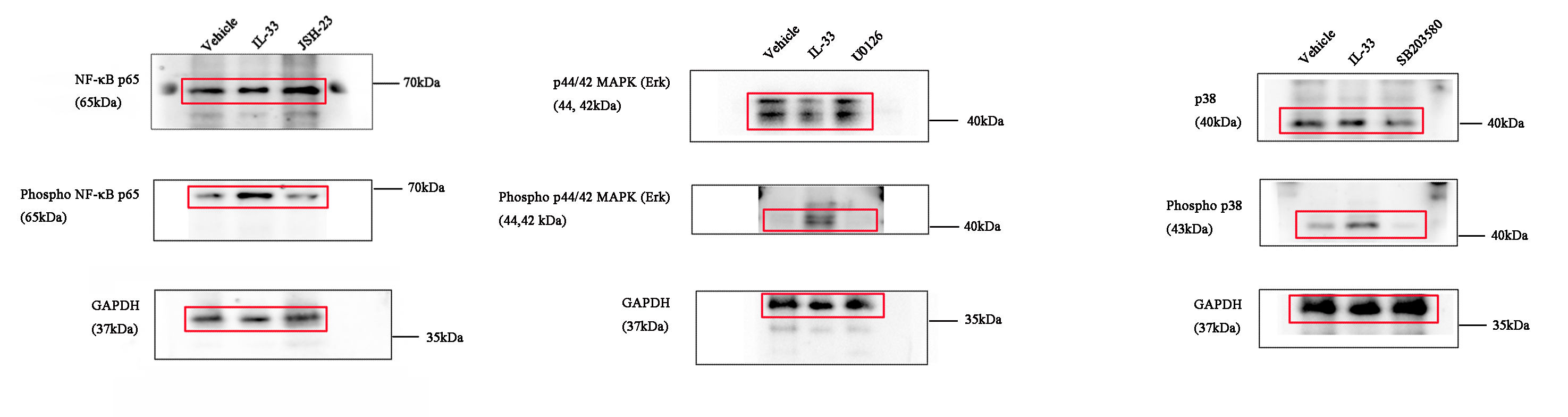

Supplement: Supplementary file 9 — Source Data Fig. 6 [file 44321_2023_12_MOESM9_ESM.zip › Figure 6/6A/6A-Western Blot.tif]

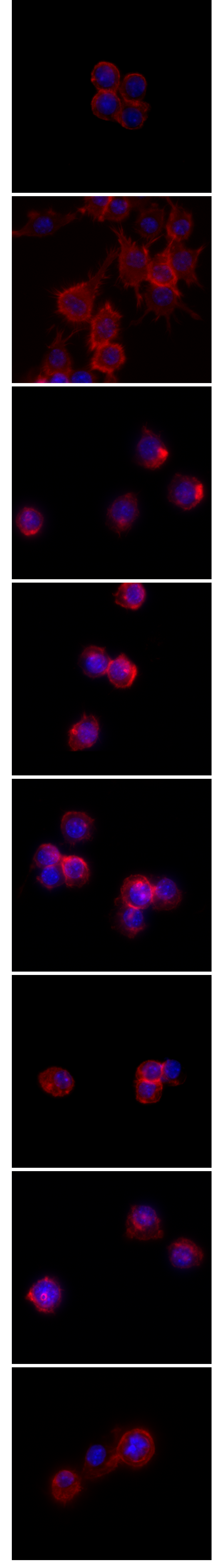

Supplement: Supplementary file 9 — Source Data Fig. 6 [file 44321_2023_12_MOESM9_ESM.zip › Figure 6/6B/6B-Immunofluorescence.tif]

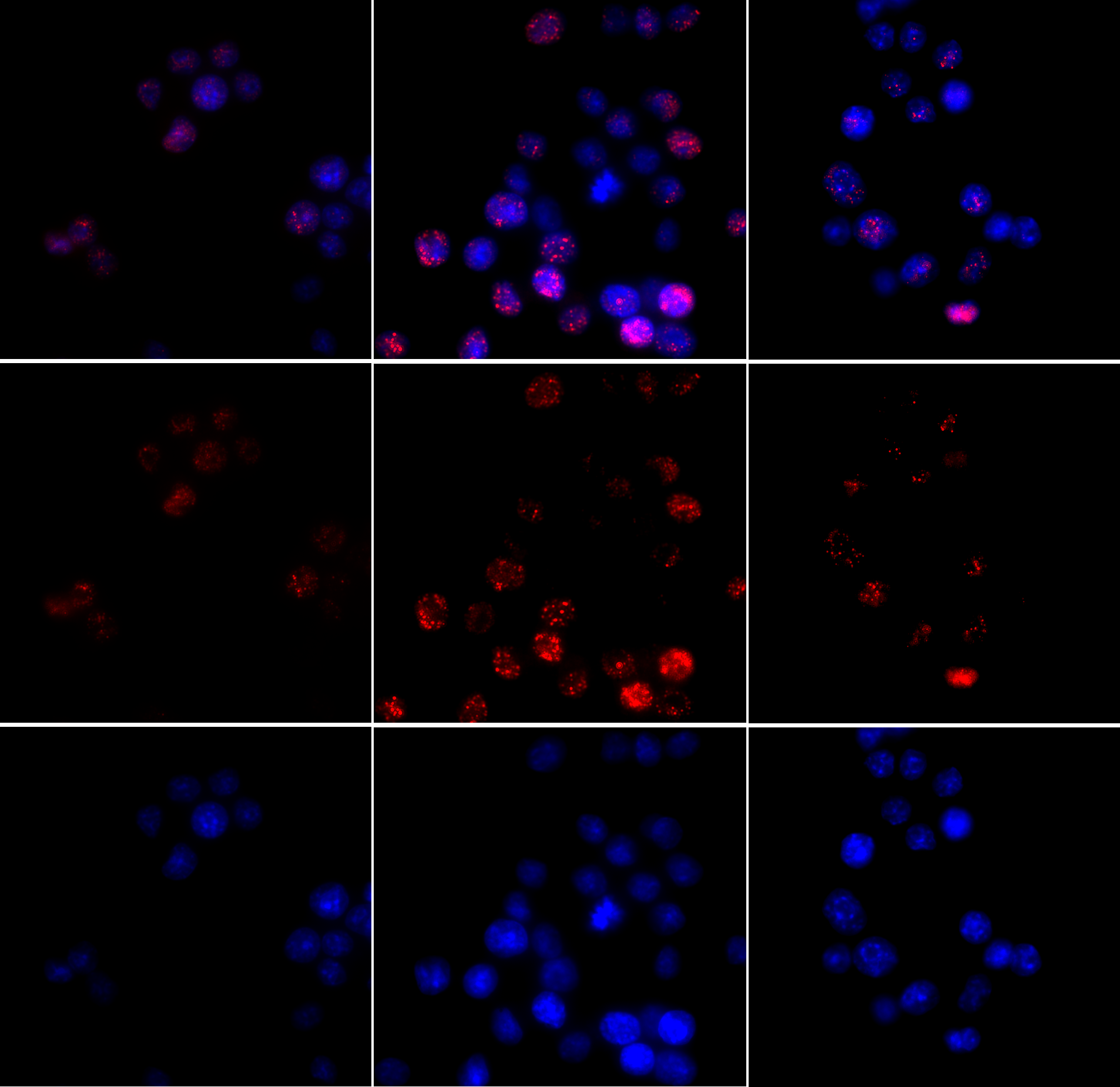

Supplement: Supplementary file 9 — Source Data Fig. 6 [file 44321_2023_12_MOESM9_ESM.zip › Figure 6/6E/6E-Immunofluorescence.tif]

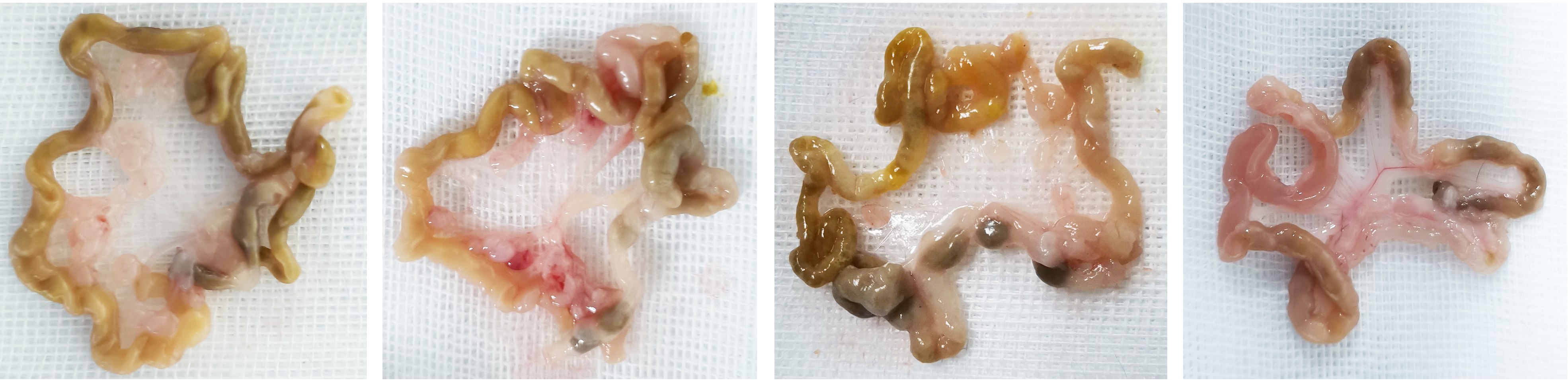

Supplement: Supplementary file 10 — Source Data Fig. 7 [file 44321_2023_12_MOESM10_ESM.zip › Figure 7/7C/7C-Mesenteric dissemination tumors.tif]

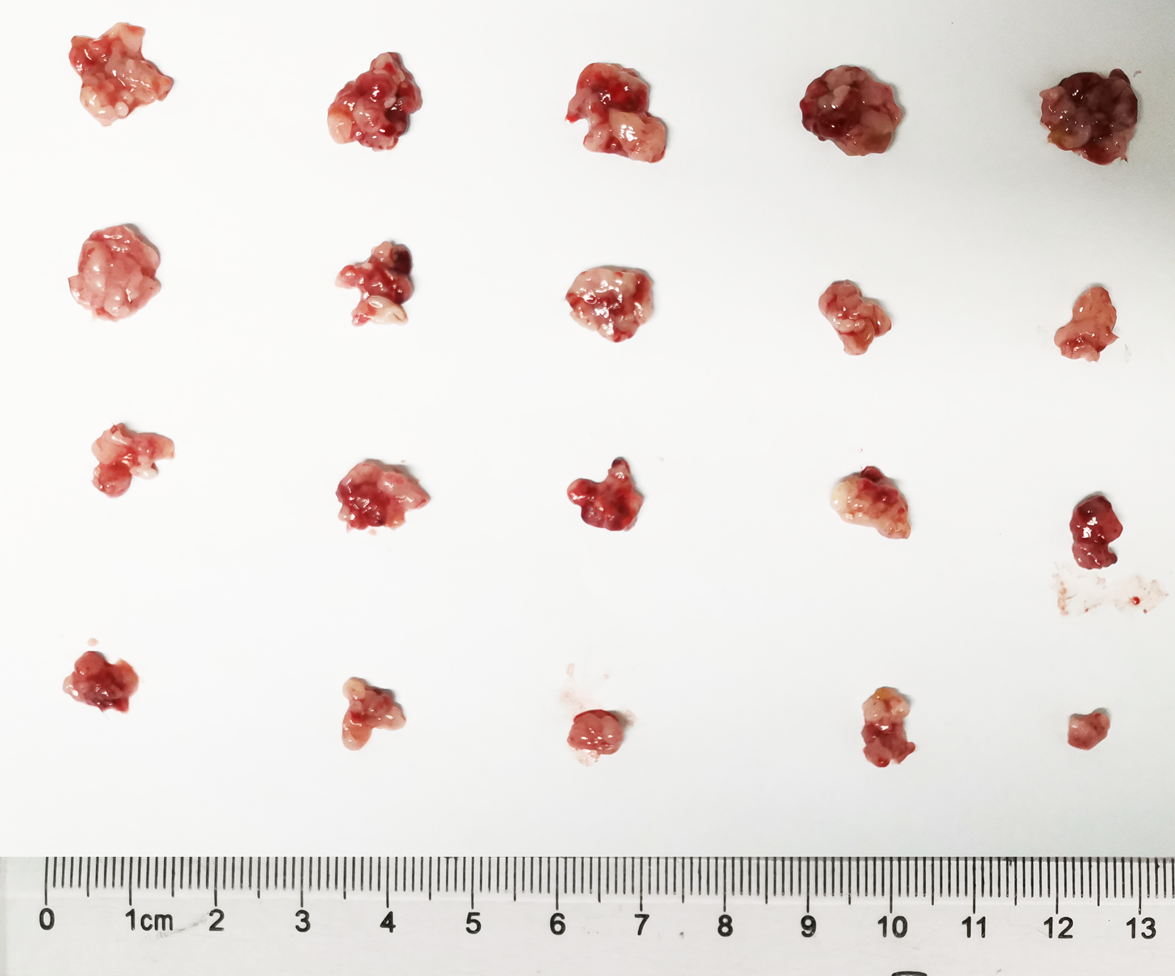

Supplement: Supplementary file 10 — Source Data Fig. 7 [file 44321_2023_12_MOESM10_ESM.zip › Figure 7/7B/7B-Abdominal dissemination tumors.tif]
